# SLEEP AND ITS TREATMENT: EFFECTS ON PHYSICAL AND PSYCHOSOCIAL WELL-BEING

**DOI:** 10.1093/geroni/igae098.0250

**Published:** 2024-12-31

**Authors:** Christopher Kaufmann, Soomi Lee, Katie Stone

**Affiliations:** Chair; Co-Chair; Discussant

## Abstract

Sleep disturbances, dysregulated circadian rhythms, and related disorders can impact multiple aspects of life, including physical health, mobility, mental well-being, and other outcomes of relevance to aging populations. Treatments for specific disorders, such as Continuous Positive Airway Pressure (CPAP) for sleep apnea, are also important for promoting positive health outcomes. Therefore, it is crucial to conduct research that clarifies the relationships among sleep, its treatments, and overall health. In this symposium, we examine the complex interplay of sleep, sleep disorders, and circadian rhythms, with physical and psychosocial well-being in older populations and their caregivers. Our first presentation uses data from the National Health and Aging Trends Study to evaluate sleep health characteristics as risk factors for physical frailty, including gender differences in this relationship. Our second presentation evaluates the influence of disrupted rest/activity circadian rhythms (measured by wrist actigraphy) on metabolic syndrome among older adult participants in the Baltimore Longitudinal Study of Aging. Our third presentation evaluates sleep quality in family caregivers and whether psychological resilience moderates the association of caregiving status with sleep quality. Our fourth and final presentation, using data from the Health and Retirement Study, identifies the comorbid chronic health conditions that may predict CPAP discontinuation in older adults with sleep apnea to identify populations requiring greater monitoring to improve treatment effectiveness. Overall, this symposium emphasizes the importance of sleep and its role in promoting positive health and well-being in aging and diverse populations.

